# Supplementary material for: Combining protein sequences and structures with transformers and equivariant graph neural networks to predict protein function
Source: Bioinformatics. 2023 Jun 30;39(Suppl 1):i318–25. doi: 10.1093/bioinformatics/btad208 (PMC10311302; doi:10.1093/bioinformatics/btad208)
Supplement: btad208_Supplementary_Data [file btad208_supplementary_data.pdf]

Supplementary Data for TransFun:

Combining protein sequences and structures with transformers and equivariant graph neural networks to predict protein function

Frimpong Boadu[1], Hongyuan Cao[2], Jianlin Cheng[1*]
[1]Department of Electrical Engineering and Computer Science, University of Missouri, Columbia, MO 65211, USA;
[2]Department of Statistics, Florida State University, Tallahassee, FL 32306, USA.
*To whom correspondence should be addressed.

Table S1. The S_{min} scores of the six single methods on the new_test_dataset. The calculation of S_{min} requires the information content (IC) of GO terms. Since some GO terms predicted by the external methods may not occur in our training dataset, we set the IC values for such GO terms to 0, which will give some advantage to the methods. Bold numbers denote the best results.

**Method**	S_{min}		
	CC	MF	BP
Naïve	14.462	17.112	44.988
Diamond	14.602	**12.388**	42.301
DeepGOCNN	14.010	14.966	42.756
TALE	13.943	13.167	43.274
DeepFri	15.197	14.956	43.175
TransFun	**12.865**	12.515	**40.528**

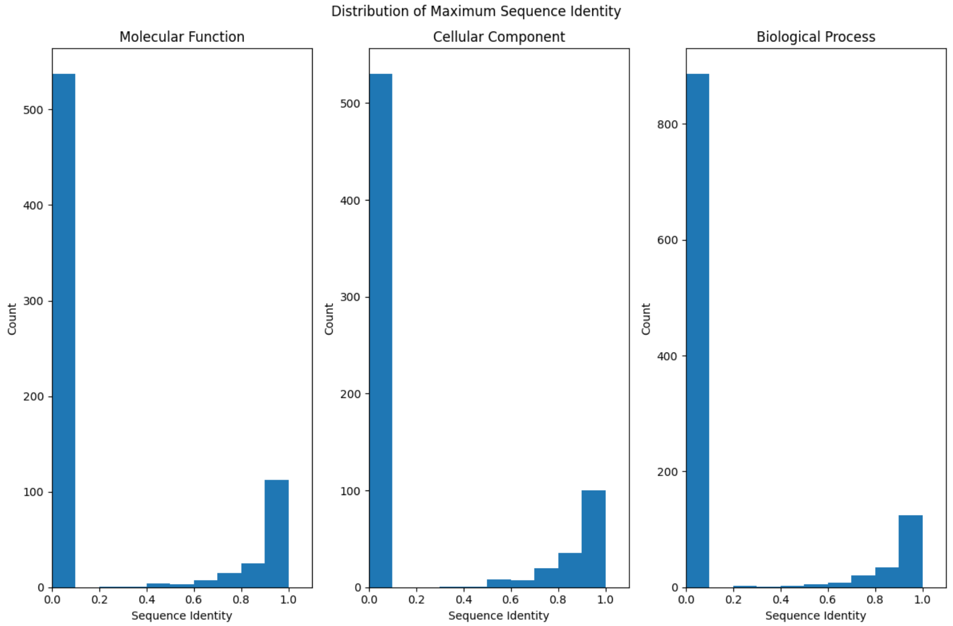

Figure S1. Distribution of the maximum sequence identity between the test proteins of three GO function categories in new_test_dataset and the proteins in the training dataset. Most test proteins have very low sequence identity (<15%) with the proteins in the training dataset.

Table S2. The results of the six individual methods on the test proteins in new_test_dataset that have less than 30% sequence identity with the proteins in the training dataset. After removing proteins with sequence identity >=30%, there are 530, 538 and 888 proteins in the cellular component, molecular function and biological process categories respectively. Bold numbers denote the best results.

Method	Fmax			AUPR		
	CC	MF	BP	CC	MF	BP
Naive	0.578	0.286	0.290	0.435	0.46	0.181
Diamond	0.406	0.341	0.271	0.080	0.118	0.071
DeepGOCNN	0.610	0.427	0.311	0.568	0.291	0.195
TALE	0.618	0.471	0.291	**0.636**	0.429	0.178
DeepFRI	0.509	0.434	0.282	0.355	0.273	0.150
TransFun	**0.634**	**0.570**	**0.303**	0.624	**0.571**	**0.297**

An ablation study of the deep learning architecture of TransFun

The architecture of the final TransFun model consists of 4 blocks of Equivariant Graph Neural Networks (EGNNs). In this section, we perform an ablation study to assess the contributions of the various EGNN blocks in the architecture and discuss the results that influence our design choices. We also use a multi-layer perceptron (MLP) that uses only sequence features as input to predict protein function. The MLP serves as a baseline to study the contribution of using protein structures to construct graph representations for the EGNN architecture of TransFun. Below are different architectural variants and designs considered in the ablation study.

EGNN1-4 denotes the final deep learning architecture used in our work. It is composed of 4 blocks of EGNNs, labeled as EGNN1, EGNN2, EGNN3 and EGNN4 respectively. Each EGNN block has 4 equivariant graph neural network layers. EGNN1 has an input dimension of 1022, equal to the feature embedding dimension for each node. It takes as input a protein graph with the per-residue embedding and generates a new embedding of dimension $C.$ C is set to the number of GO classes to be predicted. EGNN2 takes as input the protein graph and embedding features of size C from EGNN1 and produces an output of size $C/2.$ EGNN3 takes in the initial per-sequence embedding of dimension 1022 for the protein to generate the new per-sequence embedding of dimension $C/2$. The last EGNN block (EGNN4) takes as input the initial protein graph and the embedding features of dimension C/2 from EGNN2 to generate an output of dimension of C/4. The output embeddings (features) from EGNN1, EGNN2 and EGNN4 are aggregated by using a global mean pooling to obtain representative features for each protein. This is then concatenated with the per-sequence outputs of EGNN3, resulting in a $2 * C + C/4$ output features. The concatenated features are then passed through two fully connected (FC) linear layers, separated by batch normalization and RELU function to reduce the dimension to C.

EGNN-1 denotes an architecture that has the EGNN1 block but does not have EGNN2, EGNN3 and EGNN4 blocks. **EGNN1-3** denotes an architecture that has the EGNN1, EGNN2, and EGNN3 blocks, but does not have EGNN4 block. **EGNN1-2_SUM** denotes an architecture consisting of EGNN1 and EGNN2 blocks only. The input dimension of the two blocks is 1022 and their output dimension is C. The output embeddings of the two blocks are aggregated by summation instead of concatenation used in the final architecture (EGNN1-4), EGNN-1 and EGNN1-3.

The last architecture considered in this ablation study is an **MLP**, which consists of 4 linear layers, separated by a RELU activation function and dropout layers. The first linear layer takes in as input the per-

sequence embedding with a dimension of 1022 and generates an output embedding of size C, where C is the number of classes. The second layer takes in the embedding from the previous layer, with a size of C and generates an output of size C/2. This is then passed to the third layer, which takes an input of size C/2 and outputs an embedding of size C/4. We then concatenate the outputs of the layers 1, 2 and 3 as input for the fourth linear layer. The output of the fourth layer is then passed through a sigmoid layer to predict the probabilities of GO terms. Different from EGNN1-4 that use both sequence and structure inputs, the MLP uses only sequence information as input.

We compare the performance of the 5 architectures above on the validation dataset for about 10 epochs. **Figure S2** shows the F1 score of the different architectures in the three function categories (biological process, molecular function, and cellular component) on the validation dataset at a probability threshold of 0.5.

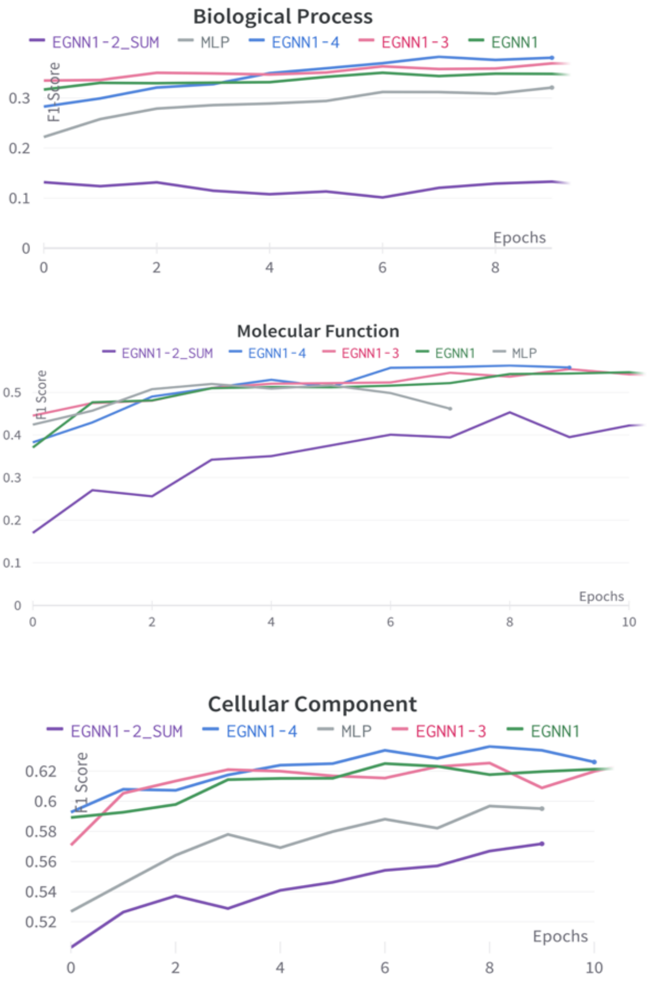

Figure S2. The F1 score of the five architectures (EGNN1-4, EGNN-1, EGNN1-3, EGNN1-2_SUM, and MLP) at the probability threshold of 0.5 on the validation dataset for about 10 epochs in the three GO function categories (biological process, molecular function, and cellular component). EGNN1-4 has four EGNN blocks. EGNN-1 has one EGNN block. EGNN1-3 has three EGNN blocks. EGNN1-2_SUM has two EGNN blocks and uses summation instead of concatenation to combine the outputs of the two blocks. MLP is a multi-layer perceptron that uses only sequence information as input without leveraging protein structures as the EGNN architectures do. Generally, the final architecture used in this work – EGNN1-4 performs better than the other architectures. Most EGNN architectures using protein structure as input

perform better than the MLP that uses only sequence information as input. EGNN1-2_SUM performs worst because it uses summation to combine the output of its EGNN blocks, while the other three EGNN architectures use concatenation to combine them.
